# Off-Label Use of Antibiotics in Small Animal Medicine—A Germany-Wide Anonymous Online Survey

**DOI:** 10.3390/antibiotics14040419

**Published:** 2025-04-21

**Authors:** Marie Tarillion, Robert Hertzsch, Angelika Richter

**Affiliations:** 1 Institute of Pharmacology, Pharmacy and Toxicology, Faculty of Veterinary Medicine, University of Leipzig, 04103 Leipzig, Germany; robert.hertzsch@tierarztpluspartner.de (R.H.); angelika.richter@vetmed.uni-leipzig.de (A.R.); 2 Tierarzt Plus GmbH, 10115 Berlin, Germany

**Keywords:** dosages, antibiotic combinations, reclassification, veterinary medicine, therapeutic success

## Abstract

**Background/Objectives:** Since 28 January 2022, veterinary medicinal products (VMPs) must be used in accordance with the conditions of the marketing authorisation (Regulation (EU) 2019/6, Article 106). This entails further restrictions on therapeutic freedom, for example, with regard to dose deviations. Off-label use is any use of a product that deviates from the Summary of Product Characteristics (SPCs). To date, there are no data available on the type and extent of off-label use on the basis of which the feasibility of the new regulation in Germany can be assessed. **Methods:** Therefore, a Germany-wide anonymous online survey was conducted as a quantitative cross-sectional study comprising 196 questions according to off-label use in dogs and cats. **Results:** In quantitative terms, the survey was representative of 358 participants but limited due to the demographic characteristics of the participants. A total of 91.3% (326/357) veterinarians stated that they had used antibiotics off-label. Fusidic acid, chloramphenicol, tylosin, and florfenicol were most frequently reclassified with regard to animal species. Authorised drugs for cats and dogs such as amoxicillin/clavulanic acid, doxycycline, metronidazole, and fluoroquinolones, like enrofloxacin and marbofloxacin, were also used off-label, often with regard to indication and treatment duration. **Conclusions:** Although there are comparatively many antibacterial preparations available for cats and dogs, off-label use is common practice. In many cases, special circumstances of the individual case justify the off-label use of authorised preparations for cats and dogs. The survey results indicate that some dose revisions are recommended. Guidance for specific indications in cats and dogs could contribute to greater legal certainty in small animal practice with regard to the justification of off-label use.

## 1. Introduction

In accordance with Regulation (EU) 2019/6 (Article 106) enacted on 28 January 2022, veterinary medicinal products (VMPs) must be used in accordance with their terms of authorisation [1]. Off-label use includes not only the deviation from the animal species and/or the indication, which is known as reclassification, but also all uses that deviate from the Summary of Product Characteristics (SPCs), such as the route of administration or dosage, which may also depend significantly on the pharmaceutical formulations of a VMP [2]. In order to avert unacceptable suffering, a reclassification may take place via the cascade provided in Article 112 of Regulation (EU) 2019/6 [1]. Considering that only drug uses outlined in the SPCs have been evaluated for a positive risk–benefit balance, justified concerns exist about the off-label use of antibiotics (ABs) with regard to therapeutic safety, efficacy, and antimicrobial resistance [3]. On the other hand, it is not only the lack of availability of authorised VMPs but also, particularly when using ABs, a lower sensitivity of causative bacteria or special features in the infection process (e.g., conditions at the source of infection) that can make off-label use necessary in order to achieve successful treatment [4]. Little is known in the literature about actual off-label use in veterinary medicine in Germany in small animals, such as dogs and cats [3]. In a non-representative survey that focused exclusively on the reclassification of anti-infectives in dogs and cats as well as other so-called small animals (e.g., rabbits) [5] and livestock [6], 67% of the 146 veterinarians surveyed reported using systemically effective ABs in a manner that deviated from the authorised animal species and/or indication [6]. In cats and dogs, systemically active ABs were used off-label in 9% of the cases. For dogs, 60% of the respondents used human medicinal products (HMPs). Moreover, 40% of veterinarians deviated from the authorised indication or used VMPs from other animal species [6]. For cats, 20% of the veterinarians reclassified HMPs and used VMPs authorised for cats for a different indication. A total of 47% of veterinarians reclassified VMPs from other animal species, and 13% chose imported preparations from other EU member states [6]. Other forms of off-label use, such as dosage deviations, were not surveyed. However, this appears important in view of current regulations within the framework of the AB minimisation concept, as underdosing and inappropriate treatment duration would be counterproductive in the effort to avoid AB resistance selection [7]. Veterinary reports on AB consumption quantities, which from 2029 will also include use in cats and dogs in the EU, will only provide limited insights into off-label use in small animal medicine. Information on reclassification in relation to the target animal species can be expected but not on deviations from dosages and indications. Likewise, pharmacovigilance based on reports of lack of efficacy of VMPs when used according to the SPCs does not provide reliable data, as such reports are rare and generally concern individual cases with limited significance [8].

It can be assumed that the doses specified in the terms of authorisation for antimicrobial VMPs are not or are no longer effective in practice for the treatment of authorised indications. Moreover, it is suspected that important indications are not covered by the SPCs. Therefore, we conducted the first comprehensive Germany-wide survey on off-label use in small animal medicine. The survey focused on the need for reclassification, as well as deviations in dosage, route of administration, and treatment duration of individual active substances from the SPCs for cats and dogs. Any deviation from the information in the SPCs, e.g., from the authorised dosage of amoxicillin of 8.75–25 mg/kg (total amount of amoxicillin + clavulanic acid in a ration 1:4), was designated as off-label use in this study. In addition, the use of non-authorised AB combinations (so-called non-fixed combinations) was also recorded according to frequency because such non-fixed combinations are criticised with regard to the selection of resistances [6]. Other topics of the survey included the prophylactic use of ABs, use for non-antibacterial purposes, and potential risks of Regulation (EU) 2019/6 for the therapeutic success of antibiotic treatments [1]. Data on off-label use in individual animal species can offer important information for the decision of which antimicrobial VMPs should be prioritised in the Europe-wide harmonisation procedure for expert information (SPC harmonisation in accordance with Article 69–72 of Regulation (EU) 2019/6) [1]. In addition, indications of a need for research into dose optimisation could be derived from this, which in turn would be important with regard to the prevention of antimicrobial resistance. In a 2017 review on the off-label use of ABs in veterinary medicine, the European Medicines Agency (EMA) called for data on the off-label use of ABs, in particular for ABs authorised exclusively for human medicine, to identify possible treatment gaps in antibacterial care in veterinary medicine [3]. This study may contribute to the assessment of potential problems arising from the authorisation process and other legal requirements, such as those currently being discussed in the context of reclassification bans [8].

## 2. Results

### 2.1. Technical Data

A total of 589 veterinarians responded to the final survey, which corresponds to a response rate of 8% in relation to the total of 7358 small animal veterinarians practising in Germany [7]. Of the 589 participants, 358 (60.8% of 589) completed the survey, and 231 (39.2%, 231/589) dropped out prematurely. For those participants who dropped out, the survey was mostly terminated while answering the demographic questions at the beginning. On average, participation in the questionnaire ceased between the questions about the non-antibacterial use of ABs (fourth block of questions, Table 1) and the questions about the use of non-authorised AB combinations (fifth block of questions, see Table 1). Of the remaining 358 participants whose answers were included in the analysis, only individual questions remained unanswered, as indicated below. Therefore, the survey results are limited in terms of representativeness. The reply included all federal states of Germany. The majority of the veterinarians who participated in the survey practised in North Rhine–Westphalia (115/355), Hesse (47/355), and Lower Saxony (43/355).

The mean survey response time was 22 min, with a median of 16 min. The survey data were checked for so-called “speeders”, i.e., participants who, by clicking through the survey quickly, have an unrealistically short response time (defined by trial runs as less than 2 min) and for so-called “straight liners”, i.e., participants who always select answers in the same position, e.g., always the first answer option [9]. In the course of the evaluation, neither “speeders” nor “straight liners” could be identified.

### 2.2. Demographic Data

Of the 358 participants, the majority of small animal practitioners stated that they were self-employed (60.1%, 215/358), worked in general practice (72.5%, 259/357), and had more than 20 years of professional experience (61.2%, 218/356). Most off-label use of ABs in relation to the proportion of patients was by veterinarians with at least 16 years of professional experience.

### 2.3. General Information on Off-Label Use

**Figure 1:**
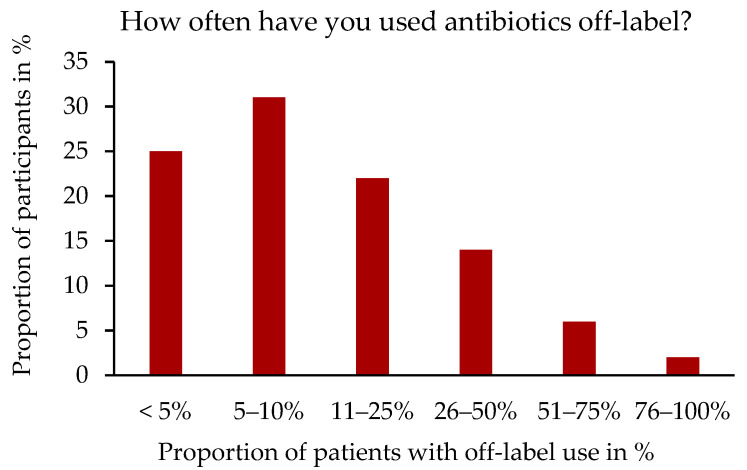
Percentage distribution of off-label use of antibiotics (ABs) in relation to the number of participants, n = 325. Single-choice question.

A total of 91.3% (326/357) of the veterinarians surveyed stated that they had used ABs off-label in the past. Moreover, 30.8% (100/325) stated that they had used ABs off-label in 5–10% of their patients, as shown in Figure 1.

The majority of these veterinarians had less than 5 years or between 16 and 20 years of professional experience. The off-label use of ABs in 11–50% of patients occurred primarily among veterinarians with 5 to 15 years of professional experience. Only veterinarians with at least 16 years of professional experience stated that they had used ABs off-label in 76–100% of their patients.

In most cases, the weight of the patients was determined as the basis for the AB dosage via weighing (88.9%, 338/344); however, in some cases, the AB dosage was determined based on the body condition score (7.4%, 28/344) and by estimation (3.4%, 13/344).

The three most common sources of information for off-label product use, such as on AB dosages, were reference books (82.1%, 294/358), package inserts (70.1%, 251/358), and guidelines (48.9%, 175/358). However, colleagues (14.5%, 52/358) and other sources of information (7.5%, 27/358) such as Vetidata, CliniPharm, or experiential knowledge were also mentioned by the participants. A total of 3% (11/358) of the participants dosed ABs on the basis of their supervisor’s dosage instructions.

The question of the most common reasons for off-label use, in general, was asked in a separate question with 10 answer options, and the participants were asked to rank these options. The evaluation was weighted using a point system. For example, the answer option in position one was weighted with a factor of ten and the answer options in the lowest position with a factor of one. The reasons for off-label product use provided by the participants were as follows in descending order: “indication” (2227 points), “duration of therapy” (1637 points), “dose” (1573 points), and “route of administration” (1434 points). Other reasons were “pharmaceutical form of the drug” (1165 points), “frequency of administration” (1110 points), “availability” (1040 points), “therapy emergency” (911 points), “package size” (790 points), and “costs” (325 points).

#### 2.3.1. Off-Label Use of ABs Authorised for Cats and Dogs

Table 1 summarises information on ABs that are available as authorised VMPs for cats and dogs in Germany. A total of 330 participants answered the question as to which active substances were used off-label. This question was independent of the frequency of general use of these antibiotics. The most frequent off-label use was for preparations containing aminopenicillin amoxicillin/clavulanic acid (68.5%, 226/330), enrofloxacin (38.8%, 128/330), and doxycycline (28.5%, 94/330), followed by metronidazole (25.5%, 84/330) and marbofloxacin (23.9%, 79/330). As summarised in detail in Table 1, the frequencies for the reasons differ for the antimicrobial substances. The main reasons were as follows: “T: Immediate treatment necessary before bacteriological test result”, “I: No VMP authorised”, “D: Insufficient effect due to non-optimal dosage according to terms of authorisation”, and “A: Administration difficulties (e.g., patient handling)”.

The main indications for off-label use were abscesses and wounds (58.2%, 192/330), infections of the urinary tract (37.6%, 124/330), respiratory tract (33.3%, 110/330), gastrointestinal tract (32.4%, 107/330), and skin and skin appendages (31.2%, 103/330), and infections of the oral cavity and teeth (30.9%, 102/330). The type of off-label use of ABs and the reasons for the particularly frequent off-label use are described in more detail in the following paragraphs.

**Figure 2:**
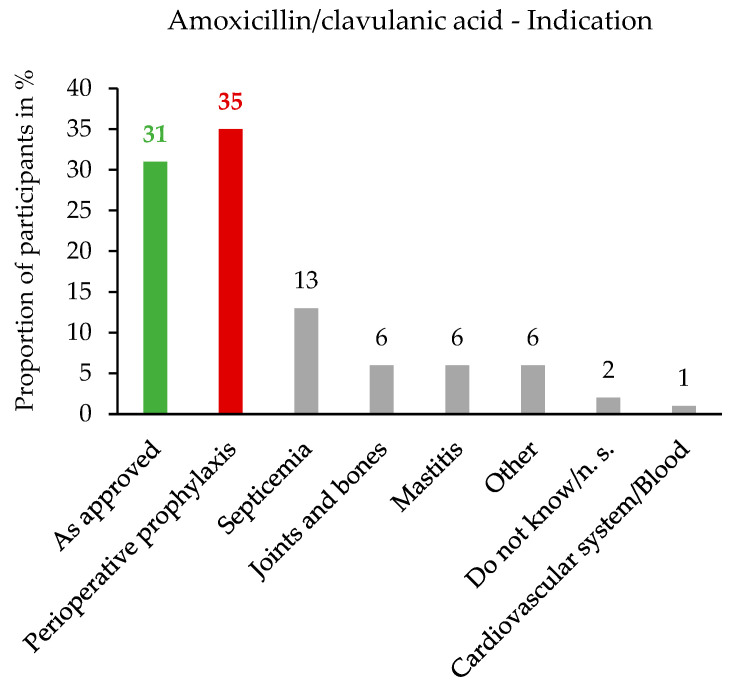
Detailed question on amoxicillin/clavulanic acid (“for what was it mainly used off-label?”): percentage distribution of indications in relation to the number of participants, n = 226. The bar on the far left indicates the authorised indications for abscesses and wounds; infections of the gastrointestinal, respiratory, and urogenital tract; infections of the skin and skin appendages; and infections of the oral cavity and teeth. The second bar from the left indicates the most frequently selected indication, perioperative prophylaxis, which deviates from the authorised indication (off-label use). Single-choice question.

The detailed question on the fixed combination of amoxicillin/clavulanic acid was answered by 226 (68.5%, 226/330) participants. At the time of the survey, twelve systemically effective amoxicillin/clavulanic acid drugs were available for cats and dogs in Germany, which were authorised for abscesses and wounds. These drugs are used for treatments of infections of the gastrointestinal, respiratory, and urogenital tract; infections of the skin and skin appendages; and infections of the oral cavity and teeth. The authorised dosage range was 8.75 to 25 mg/kg based on the total amount of active substances (amoxicillin/clavulanic acid in a ratio of 4:1) for oral and intramuscular administration. The authorised duration of treatment was five to seven days or longer for chronic or severe cases. Based on the survey question results, off-label use was often based on the indication (Figure 2).

Although the authorised dosage of amoxicillin/clavulanic acid was predominantly used by 80.1% (181/226) of the participants, 15.0% (34/226) used a higher dosage than authorised—25.1–37.5 mg/kg and 1.0% (3/226) at 37.6–50 mg/kg—regarding amoxicillin/clavulanic acid. In addition to the authorised administration frequency of once or twice daily, amoxicillin/clavulanic acid was also administered three times daily by 17.9% (40/223) and more than three times daily by 1.3% (3/223) of the participants. In addition to the authorised oral, subcutaneous, and intramuscular routes of administration, the veterinarians stated that they had also administered the combination intravenously (13.4%, 30/224), presumably after reclassification with regard to the animal species or after reclassification of a human drug. Intravenous administration was the first choice for septicemia (12/30), followed by the prevention of surgical infections (6/30). Amoxicillin/clavulanic acid was used longer than authorised by 12.9% (29/225) of the participants. The most common reason provided for off-label use of amoxicillin/clavulanic acid was “Immediate treatment required before bacteriological test result” at 38.5% (87/226), followed by “Insufficient effect due to non-optimal dosage according to terms of authorisation” at 28.3% (64/226) and “Administration difficulties”, e.g., with regard to patient handling, at 23.9% (54/226). A total of 30 of the 64 veterinarians who indicated the reason for an ineffective dose was according to the terms of authorisation dosed higher than authorised with up to 50 mg/kg of the total amount of active substance. Moreover, 22 of these veterinarians (22/64) administered the combination more frequently, up to more than three times daily, and 9 of these veterinarians (9/64) used amoxicillin/clavulanic acid longer than authorised.

Enrofloxacin was used off-label by 38.8% (128/330) of the participants. At the time the survey was conducted, there were 14 systemically effective enrofloxacin drugs for dogs and 9 for cats for the treatment of abscesses and wounds, infections of the gastrointestinal, urogenital, and respiratory tracts, and infections of the skin and skin appendages for oral and subcutaneous administration with an authorised dose of 2.5–5 mg/kg per single dose. The authorised duration of treatment was 5 to 10 days; however, treatment can be extended to up to 49 days, in the case of deep pyoderma in dogs, for example. Overall, several participants chose off-label uses for enrofloxacin, with the authorised indication (36.5%, 46/126) being followed by the off-label indications of “septicemia” in 22.2% (28/126) and “other” in 17.5% (22/126) of the participants. Topical administrations on the ear and eye and on pets such as rabbits were mentioned under “other”. However, topical administrations on species other than cats and dogs were explicitly excluded from the survey. The use of enrofloxacin in septicemia, which deviated from the approved SPC, was largely due to the need for immediate treatment before the bacteriological test result (18/21). Usually, the authorised dosage was used, but ten (8.0%, 10/125) veterinarians dosed enrofloxacin higher, and two (1.6%, 2/125) veterinarians used a lower dose. Ten veterinarians chose the answer option “other”. The associated free-text comments mainly included information on other animal species and dosages for topical administrations, which were specifically excluded from this survey. For cats, it was indicated that enrofloxacin was used for longer periods by 26.7% (32/120) of veterinarians in addition to the recommended treatment duration of 5–10 days (63.3%, 76/120). Reasons for off-label use of enrofloxacin were, in descending order, the need for immediate treatment prior to bacteriological test results (37.5%, 48/128), no authorised VMP (22.7%, 29/128), mixed infections (21.9%, 28/128), and administration difficulties (21.1% (27/128), e.g., in relation to patient handling.

Doxycycline was used off-label by 28.5% (94/330) of the survey participants. At the time of the survey, two doxycycline drugs were authorised for cats and dogs in Germany. The authorised indication for dogs includes urogenital and respiratory tract infections and for cats respiratory tract infections with an authorised dosage of 5–10 mg/kg each for 5 to 10 (cats) or 14 days (dogs). In the survey, off-label use for dogs predominated in terms of indication and treatment duration. Doxycycline was most frequently used off-label for antiparasitic treatment (42.6%, 40/94). In second place was the response option “other” with 21.2% (19/94). Among the associated free-text comments were mainly treatments of vector-borne diseases such as anaplasmosis, borreliosis, ehrlichiosis, or leishmaniasis and, in addition, indications such as immunomodulation, the treatment of eye diseases or the treatment of infections associated with anaemia and fever, and the treatment of leptospirosis. The authorised indication was only in third place with 8.5% (8/94). In cats, the authorised indication predominated with 32.2% (29/90) but was closely followed by the off-label indication “other” with 18.9% (17/90) and “for infections of the oral cavity and teeth” with 12.2% (11/90). Under “other”, chlamydia-related eye infections, anaplasma, babesia, hemobartonella, mycoplasma infections, immunomodulation, infections with high fever and anaemia, and pododermatitis were mentioned in the form of free-text comments. A total of 54.3% (51/94) of the veterinarians stated that they had used doxycycline for dogs longer than authorised. The main reason provided for the off-label use of doxycycline was “No VMP authorised” with 35.1% (33/94), which was mostly due to the deviating indication for antiparasitic therapy in dogs (25/33). This was followed by “no sufficient effect due to non-optimal dosage according to the terms of authorisation” with 29.8% and “other” with 16% (15/94). Free-text comments that can be summarised under the generic term “source of information on the use of AB” such as “treatment recommendation in the literature”, “recommendations from specialists”, and “specialist information given during scientific conferences and from colleagues that do not correspond to the package leaflet” were included under “other”. Other comments were in favour of a longer treatment duration than authorised in order to achieve the desired therapeutic success. Further comments noted poor tolerability of the authorised doxycycline VMPs compared with the human formulation. This was expressed, for example, by vomiting after administration of the VMP. In addition, individual veterinarians stated that doxycycline is used to treat mycoplasma infections, although this exceeds the authorised area of indication of the authorised VMPs available.

A total of 25.5% (84/330) of the veterinarians stated that they had used metronidazole off-label. At the time of the survey, five drugs for dogs and four for cats were authorised in Germany for infections of the gastrointestinal tract due to *Giardia* spp. and *Clostridia* spp., urogenital tract, and skin and skin appendages, as well as for infections of the oral cavity and teeth with a dosage of 25–50 mg/kg per single dose for oral administration for five to seven days. Due to off-label use, the duration of treatment was particularly striking. The votes were equally divided between the authorised duration of five to seven days (46.9%, 38/81) and a longer treatment duration than authorised (46.9%, 38/81). The majority of veterinarians (59%; 49/83) stated that they had used metronidazole as authorised at 25–50 mg/kg per single dose. More than a third of the participants chose a lower dosage: 25.3% (21/83) chose 12.5–24.9 mg/kg per single dose, 12.1% (10/83) < 12.5 mg/kg per single dose, and one free-text comment also stated that the dosage was lower. In terms of the route of administration, 18.3% (15/82) of the veterinarians stated that they had also used metronidazole intravenously instead of orally as authorised (80.5%, 66/82), presumably after reclassification due to animal species or after reclassification of a human drug. The reasons for off-label use of metronidazole were, in descending order, lack of efficacy due to the dosage specified in the terms of authorisation (26.2%, 22/84), no authorised VMP (23.8%, 20/84), and “other” (16.7%, 14/84). Various free-text comments were formulated by the veterinarians in connection with “other”: five veterinarians stated that infection with giardia was not sufficiently treated with fenbendazole. Treatment in combination or alone with metronidazole is the most effective, according to comments from individual veterinarians. Some participants stated that a lower dosage than specified in the terms of authorisation was completely sufficient and suggested 15 mg/kg for infections of the gastrointestinal tract according to specialist books, publications, and personal experience. Three comments suggested that a longer duration of therapy of more than seven days or continuous medication was sometimes necessary. The use of a lower dosage than authorised (31 participants; <12.5 mg/kg: 10 participants; 12.5–24.9 mg/kg: 21 participants) was not based on poor tolerance. Ten of thirty-one veterinarians used lower doses of metronidazole for antiparasitic infections, one veterinarian for septicemia (1/31), one for cholecystitis (1/31), and one for respiratory tract infections (1/31).

A total of 24% (79/330) of all participating veterinarians stated that they had used marbofloxacin off-label. At the time of the survey, six drugs were licensed for cats and eight for dogs with a dosage of 2 mg/kg for cats and 2–4 mg/kg for dogs in Germany. Indications covered by the terms of authorisation for cats included abscesses and wounds, infections of the skin and skin appendages, infections of the respiratory tract, and the prevention of surgical infections for three to five days. For dogs, the indications of infections of the urogenital tract were also authorised. The authorised duration of treatment for dogs was 5 days and up to 40 days in severe cases of infection of the skin and soft tissue. In addition to the authorised indication (47%, 36/76), marbofloxacin was often used in dogs for septicemia (20%, 15/76) and for infections of joints and bones (11%, 8/76). The use of marbofloxacin for the treatment of septicemia was almost exclusively (12/15) due to the need for immediate treatment prior to the bacteriological test result. In cats, off-label use predominated in terms of indication due to its use in urinary tract infections (35%, 26/76), septicemia (11%, 8/76), and infections of the oral cavity and teeth (10%, 7/76). In cats, marbofloxacin was also dosed higher at 3.1–4 mg/kg (24%, 18/76). In cats, marbofloxacin was used for longer periods (39%, 30/77) than authorised (three to five days or prophylactically once). The most common reason provided for the off-label use of marbofloxacin was the need for immediate AB treatment before the bacteriological test result (52%, 41/79), followed by “administration difficulties”, e.g., inappetence when administering tablets (34%, 27/79), and “insufficient effect due to non-optimal dosage according to the terms of authorisation” (24%, 19/79).

A total of 23% (76/330) of the veterinarians stated that they had used cefovecin off-label. At the time of the survey, only one VMP with an authorised dosage of 8 mg/kg was authorised for cats and dogs as a subcutaneous one-shot preparation in Germany. In the case of severe infection of the skin and soft tissues, the administration can be repeated up to three times in dogs and once every 14 days in cats. For cats, the indications of abscesses and wounds, infections of the skin and skin appendages, and infections of the urogenital tract were covered by the terms of authorisation. In dogs, infections of the oral cavity and teeth were also authorised as an indication. The survey data show that cefovecin was also used in dogs for infections of the respiratory tract (9.5%, 7/74) and “other” (9.5%, 7/74), in addition to the authorised indication. “Other” included various comments, such as that cefovecin was not yet used in dogs or that it was used for other animal species. Other free-text comments were “eye”, “urinary tract”, and “pancreatitis”. “Eye” falls under the category of topical administration of ABs, which was excluded from this survey, as mentioned several times in the questionnaire. The other free-text comments can be assigned to the response options that were available to the participants. In cats, cefovecin was mainly used off-label for the treatment of infections of the oral cavity and teeth (32%, 24/75) and for infections of the respiratory tract (17.3%, 13/75). In 45 of 55 cases, the deviating indication in cats was due to administration difficulties, which was presumably due to the long-term effectiveness of the VMP 14 days after only one administration, according to the terms of the authorisation. In addition to the authorised duration of treatment, cefovecin was also used for longer periods (43.4%, 33/76) in cats and dogs. The most common reason provided for off-label use was “administration difficulties”, e.g., patient handling (80.3%, 61/76), followed by the need for immediate treatment before the result of a bacteriological examination (38.2%, 29/76) and “other” (14.5%, 11/76). Under “Other”, various reasons were provided in the form of free-text comments, which are shown in Table 2.

#### 2.3.2. ABs Authorised for Other Animal Species

Among the antimicrobial substances authorised for systemic therapy of other animal species for which there are no authorised VMPs for cats and dogs (Table 3), fusidic acid, chloramphenicol, tylosin, and florfenicol were frequently reclassified. Tetracycline, ronidazole, polymyxin B, which is only authorised for local use as a VMP, and orbifloxacin were also among the most frequently reclassified ABs.

A few veterinarians (1–10) also used the following ABs in descending order: kanamycin, tulathromycin, ceftiofur, chlortetracycline, cloxacillin, oxacillin, carnidazole, colistin, furazolidone, bacitracin, cefoperazone, dihydrostreptom, apramycin, dimetridazole, penethamate, tiamulin, tildipirosin, and tilmicosin. In order to limit the overall scope of questions, no questions were asked about patient proportions. Reasons for reclassification resulted from the lack of availability of authorised drugs for the indication.

#### 2.3.3. Reclassification of ABs of HMPs

A total of 349 out of 358 participants answered the question about the relevance of antimicrobial HMPs in small animal medicine. A total of 47.9% of the participants stated that they had previously reclassified antimicrobial HMPs. This took place mainly in less than 1% of patients (55.4%, 92/166), in 29.5% (49/166) in 1–5% of patients, and in 10.2% (17/166) in 6–25% of patients. The main reasons for the reclassification of human ABs were the formulation of the product (49.1%, 82/167), problems with availability (44.3%, 74/167), indication (19.2%, 32/167), and dosage strength (16.2%, 27/167). With regard to the large number of anti-infectives used in human medicine, questions according to the reclassified ABs were not asked because the list of answer options would have been too long. Instead, the survey asked the question of whether the Implementing Act (EU) 2022/1255 [10], by which certain ABs are reserved exclusively for human medicine, poses therapeutic problems for cats and dogs. According to the survey, the ban on the use of these active substances in small animal medicine does not cause therapeutic problems for 79.3% (275/347) of the participants.

#### 2.3.4. Prophylactic and Non-Antibacterial Use

**Figure 3:**
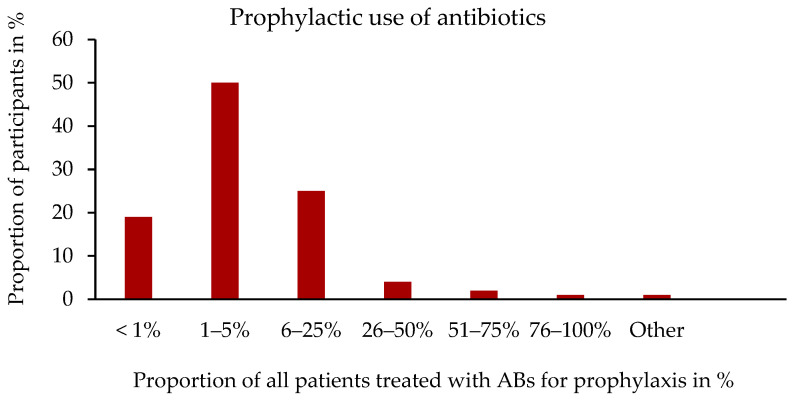
Frequency of prophylactic use of ABs. Percentage distribution in relation to the number of participants, n = 139. Single-choice question.

The question about the prophylactic use of ABs was answered by 353 of the 358 participants. A total of 39.4% (139/353) of the participants stated that they had used ABs prophylactically. Of these, 49.6% (69/139) stated that they had used ABs in 1–5% of patients (Figure 3).

The most common indications that led to prophylactic use were generally for surgery (49.6%, 69/139), other (31.7%, 44/139), and for vulnerable geriatric patients (29.5%, 41/139). Numerous free-text comments were mentioned under “Other”, which are summarised in Table 4.

**Figure 4:**
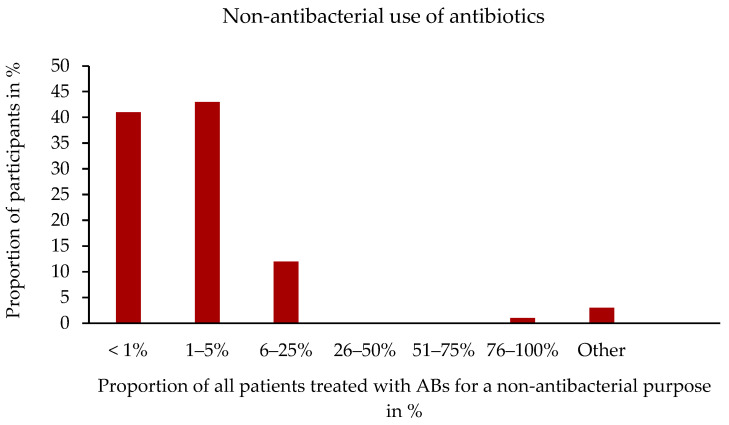
Non-antibacterial use of antibiotics by frequency. Percentage distribution in relation to the number of participants. Other: “In giardia-positive animals”; “Coccidiosis”, n = 76. Single-choice question.

A total of 350 of the 358 participants answered the question about the use of ABs for non-antibacterial purposes. A total of 22.3% (78/350) stated that they had already used ABs for non-antibacterial purposes. Of these, 43.4% (33/76) used ABs in 1–5% of patients and 40.8% (31/76) in less than 1% of patients for non-antibacterial purposes (Figure 4). ABs were used for AB-responsive diarrhoea (56.4%, 44/78) and immunomodulation (53.9%, 42/78) and for their antiparasitic (48.7%, 38/78), anti-inflammatory (14.1%, 11/78), or prokinetic effects (3.9%, 3/78). The response option “Other”, which is linked to a free-text comment, was also selected by 10.3% (8/78) of veterinarians. These included free-text comments, such as “viral cat flu”, “cystitis”, “resistant Giardia strains”, “improvement of corneal collagen cross-linking”, and “coccidiosis”.

#### 2.3.5. Use of Non-Fixed AB Combinations

**Figure 5:**
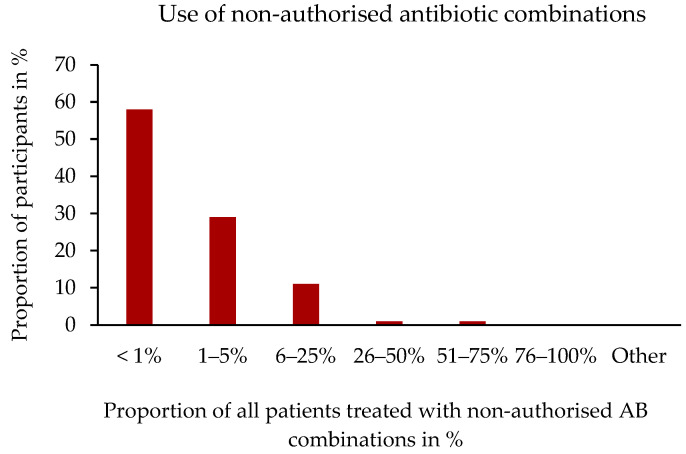
Use of non-fixed antibiotic combinations by frequency. Percentage distribution in relation to the number of participants, n = 258.

A total of 72.0% (254/353) of the participants reported having used two, and 19.0% (67/352) reported having used more than two different ABs together that were not already included as a fixed combination as an authorised VMP. Moreover, 57.8% (149/258) of these participants used those AB combinations in less than 1% of their patients and 29.1% (75/258) in 1–5% of their patients (Figure 5).

According to the survey, the three most frequently used combinations were amoxicillin/clavulanic acid combined with fluoroquinolones (82.95%, 214/258), metronidazole (36.1%, 93/258), or doxycycline (15.9%, 41/258), as summarised in Table 5. “Others” included combinations of amoxicillin/clavulanic acid + fluoroquinolones + metronidazole (n = 4), amoxicillin + fluoroquinolone (n = 3), amoxicillin/clavulanic acid + cephalosporins (n = 3), and penicillin + gentamicin (n = 2).

Combinations were mainly used for sepsis (57.4%, 148/258) and acute systemic infection to overcome the time until the antibiogram result (56.2%, 145/258), aspiration pneumonia and other respiratory tract infections (41.1%, 106/258), or wound healing disorders (32.6%, 84/258). The main reasons for the use of these non-authorised combinations were the need for immediate treatment before receiving the bacteriological diagnosis (61.6%, 159/258), administration difficulties (e.g., patient handling or oral treatment in an animal with vomiting or inappetence) (51.2%, 132/258), an insufficient effect due to inadequate authorised dosages (45.7%, 118/258), and the lack of an authorised VMP (41.5%, 107/258).

### 2.4. Therapeutic Success Through Off-Label Use and Effects of the New Regulation

**Figure 6:**
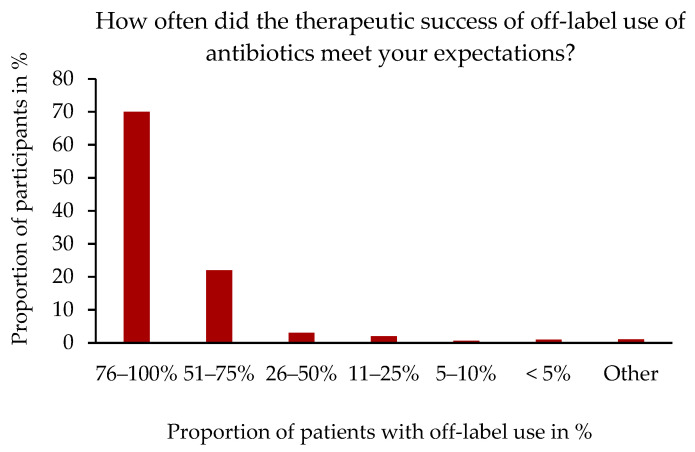
“How often did the therapeutic success of off-label use of antibiotics meet your expectations?” In % of cases in relation to the number of participants, n = 329. Single-choice question.

A total of 329 of the 358 participants answered the question about the therapeutic success of off-label use of ABs. Moreover, 70.5% (232/329) stated that the therapeutic success of the off-label use of ABs met their expectations in 76–100% of cases and 22.3% (73/329) in 51–75% of cases (Figure 6).

In addition, the survey asked the question of whether the participants consider the success of the therapy to be at risk as a result of Regulation (EU) 2019/6, Article 106 (1) [1], i.e., “Veterinary medicinal products shall be used in accordance with the terms of marketing authorisation”. The question was answered by 356 participants. A total of 72% of the participants consider the success of treatment to be “at risk” or “at high risk” if this regulation is strictly adhered to.

**Figure 7:**
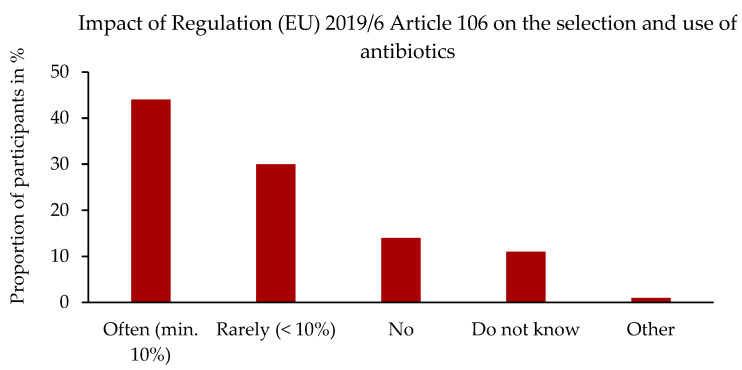
“Does the new regulation have an influence on your choice and use of antibiotic preparations compared with the time before the new legal situation/before 28.01.2022?” Relative frequency in percent in relation to the number of participants, n = 356. Single-choice question [1].

Figure 7 shows the distribution of responses to the question of whether the new regulation has an impact on the selection and use of AB preparations.

In a separate question, participants were asked to indicate with “yes” or “no” whether their off-label use had changed as a result of the new legal situation (not illustrated). A total of 46.2% (162/351) stated that nothing had changed in their off-label use since the new legal situation. Moreover, 152 of the 351 participants explained their decision in the free-text comments, indicating problems in the storage of too many VMPs, adequate route of administration, and insufficient doses. Some veterinarians stated that they feel challenged by the new regulation, i.e., in relation to the need to achieve a balance between the best possible treatment of their patients and to act in a manner consistent with the law.

## 3. Discussion

To the best of our knowledge, this is the first survey conducted in an EU country that provides comprehensive data on the actual off-label use of ABs in small animal medicine since the introduction of Regulation (EU) 2019/6 [1].

Considering the number of participants, the present survey can be considered representative, but as a limitation, some questions remained unanswered by the participants. Demographic data showed that most participants worked in the field of general small animal medicine, had at least 20 years of practical experience, and were self-employed. This group probably feels more “threatened” by the new regulation or restricted in their freedom of therapy and, therefore, has a greater interest in the topic than, for example, employed veterinarians with little professional experience. This may explain the above-mentioned majority of the participants. Due to the lack of available comparable demographic data, the qualitative representativeness of the surveyed veterinarians was not verified. Nevertheless, the results of this survey provide insights into aspects of off-label use that will not be addressed via the future recording of AB consumption in cats and dogs in accordance with Article 57 of Regulation 2019/6 [1].

The results substantiate that off-label use of ABs is common practice in small animal medicine, as 91.3% (326/357) of the participants stated that they had used ABs off-label in the past. As shown in the present survey, the most frequent types of off-label use, including reclassification of VMPs or HMPs, are “indication”, “therapy duration”, “dose”, and “route of administration”, as discussed in the following sections. From the detailed questions on individual ABs authorised for cats and dogs, the following reasons were predominant: “Immediate treatment necessary before bacteriological test result”, “No VMP authorised for the indication”, “No sufficient effect due to non-optimal dosage according to the terms of authorisation”, and “administration difficulties”.

Authorised doses of older antimicrobial products have not been reviewed for a long time, while veterinary pathogens may have undergone changes in their susceptibility to AB, which may require dose adjustments [4]. Pharmacovigilance is a very effective tool to identify insufficient efficacy of VMPs [8], but this would require a dramatic increase in reports by veterinary practitioners. Therefore, this survey mainly focused on the off-label use of ABs authorised for cats and dogs to obtain information about the need to review dosages, e.g., due to a project on dose revision (ADRA) by pharmaceutical companies. The harmonisation of the Summary of Product Characteristics (SPCs), which is outlined in EU Regulation 2019/6 (Article 70–71) [1], does not offer the possibility of making changes to content such as dose adjustments. This EU-wide regulation is purely a formal process.

### 3.1. Off-Label Use of Authorised Products Concerning Indication

Considering the large number of authorised products available according to the German database (www.vetidata.de), the frequent off-label use of ABs in terms of indication was unexpectedly high, especially for the fixed combination amoxicillin/clavulanic acid and for doxycycline. Amoxicillin/clavulanic acid was frequently used for perioperative prophylaxis. Indeed, there were no antimicrobial VMPs for this indication at the time of the survey. In view of the broad spectrum of activity against Gram-positive, Gram-negative, and beta-lactamase-producing bacteria [11,12], its use for the prevention of surgical infections seems reasonable in several cases, as discussed below (see Section 3.4). On the other hand, perioperative antimicrobial prophylaxis, including elective surgery, is viewed critically by the EMA with regard to the low incidence (0–0.9%) of all postoperative infections in elective procedures, such as arthroscopy [3].

Doxycycline stood out in terms of the treatment of parasitosis, such as anaplasmosis, borreliosis, and ehrlichiosis. Doxycycline also has a broad spectrum of activity for protozoa, rickettsia, and ehrlichia [13]. Its use in vector-borne disease is described in the literature (see below) [13]. However, the preparations available for cats and dogs are only authorised for infections with *Bordetella bronchiseptica*, *Pasteurella* spp., *Leptospira* spp. (dogs only), and *Chlamydia felis* (cats only). There is a therapeutic gap with regard to guidelines for the treatment of vector-borne disease in small animal medicine. Furthermore, the American Heartworm Association recommends doxycycline because of its effect against endosymbiotic Wolbachia as an add-on therapy for heartworm infections [13,14]. Based on the international trade in cats and dogs and travel, those diseases are increasingly observed in Germany [15]. As supported by our survey, there is a need for VMPs with doxycycline that cover indications that have become increasingly common in countries such as Germany.

The fluoroquinolones enrofloxacin and marbofloxacin, which are authorised for cats and dogs, were used off-label by many participants, often in cases of septicemia with the need for immediate treatment. As described in the literature [13], marbofloxacin was also used for joint and bone infections in dogs and for urinary tract infections in cats. Furthermore, enrofloxacin is also effective against rickettsiae in dogs, and marbofloxacin is effective against intracellular *Mycoplasma haemofelis* in cats [13]. Overall, the survey results demonstrate that fluoroquinolones are used for serious illnesses such as septicemia or joint and bone infections when lower-class ABs have been exhausted or there is imminent danger. This is in line with the AMEG’s recommendations on the use of these important active substances. As ABs from category B, according to the AMEG, fluoroquinolones should only be used very restrictively and only if first-line ABs (category D) and second-line ABs (category C) are ineffective.

As in the case of cefovecin, the main reason for off-label use concerning the indication was the easier administration, i.e., a single injection to achieve a 14-day duration of action [16]. Indeed, daily oral treatment can be difficult for pet owners, especially in the treatment of cats, leading to insufficient compliance. Therefore, there is a need for more sustained release formulations of first- and second-line ABs. It should be noted that cefovecin should not be used off-label for purely practical reasons; however, treatment with “reserve” antibiotics deserves careful diagnosis.

### 3.2. Off-Label Use of Authorised Products Concerning Dosing Instructions

Many participants used amoxicillin/clavulanic acid at higher doses, up to a daily dose of 25.1–50 mg/kg of amoxicillin, and shortened the dosing intervals or prolonged the treatment duration with the reason of “No sufficient effect due to non-optimal dosage according to the terms of authorisation”. This indicates that a review of dosages is reasonable. Higher doses are at least recommended for infections with Gram-negative pathogens [12,13].

In contrast, several participants used metronidazole at lower doses (<12.5 mg/kg), which could not be traced back to poor tolerance. Some of the free-text comments indicated that the participants considered a dose of metronidazole lower than the authorised dose to be sufficiently effective. This is in line with recommendations in the literature for specific indications, e.g., doses up to 10 mg/kg for the treatment of inflammatory bowel diseases or 7.5 mg/kg for the therapy of hepatic encephalopathy in dogs [17,18].

### 3.3. Reclassification of Antibiotics Not Authorised for Dogs and Cats and the Use of the Non-Fixed Combinations

When the survey was conducted, there were more than 20 antimicrobial substances authorised for cats and dogs in a total of 74 (cats) and 105 (dogs) systemically effective VMPs on the market in Germany (VETIDATA, as of 13 July 2022). Thereby, many classes of ABs were available, including penicillins, tetracyclines, aminoglycosides, potentised sulfonamides, nitroimidazoles, lincosamides, macrolides (for dogs only), fluoroquinolones, quinolones, and first- and third-generation cephalosporins as well as authorised (fixed) combinations (amoxicillin/clavulanic acid, aminoglycoside/lincosamide, and macrolide/nitroimidazole). Therefore, it was unexpected that many participants would state that they had reclassified antimicrobial VMPs and HMPs for cats and dogs. In general, reclassification is associated with increased uncertainties, as recommendations of dosages described in the literature are generally related to the active substance and cannot take into account the pharmaceutical formulations of a medicinal product. The risks of off-label use are difficult to assess; however, studies in the UK showed that 7% of adverse events were associated with off-label use [19].

In our survey, which mainly focused on off-label use of authorised VMPs for dogs and cats, the extent of reclassification related to all patients was not considered; however, 330/359 veterinarians stated that they had reclassified VMPs. In contrast, in a previous less comprehensive survey with a low response rate, only 9% of German veterinarians stated that they had reclassified antimicrobial VMPs in small animals [6]. In favour of the response rate, the overall scope of the questions of our survey was limited, and questions regarding possible legal discrepancies were avoided. Therefore, the frequency of use in relation to patients, specific reasons, and the performance of bacteriological tests for reclassification of VMPs were not asked. Indeed, more specific ongoing surveys concerning indications [20] or bacteriological diagnostics [21] should provide more insights into the reasons. Furthermore, it can be expected that the extent of reclassified VMPs and HMPs will become apparent through the collection of data on antimicrobial medicinal products used in dogs and cats according to Regulation (EU) 2019/6, Article 57 [1], and German law.

In line with previous surveys [3], almost half of the participants stated that they had reclassified antimicrobial HMPs in a small percentage of their patients. With regard to the large number of antimicrobial substances that are authorised as HMPs, this survey did not ask which of them were reclassified. Another German study showed that HMPs were only used in 4.4% of treatments [22]. The majority of HMPs were used for topical therapy (95.0%), e.g., as eye or ear drops. The main drugs used were gentamicin (63%), ofloxacin (13%), and oxytetracycline (8%) [22]. Depending on the substance and the availability of authorised drugs in different countries, the reclassification is probably globally highly variable. As mentioned above, data on the reclassification of HMPs in the individual EU states are expected by the implementation of Article 57 of Regulation (EU) 2019/6 [1]. Based on the Implementing Act (EU) 2022/1255 [10], several antimicrobials, such as carbapenems, are reserved for the treatment of certain infections in humans. Notably, the majority of the participants of the present survey (77%) stated that the ban on these ABs does not cause any therapeutic problems.

According to guidelines on the prudent use of ABs in animals to prevent the selection of antimicrobial resistance, the use of non-fixed combinations of antibacterial drugs should be avoided [7]. However, our survey indicates that this is common practice in small animal medicine. Popular combinations were amoxicillin/clavulanic acid with fluoroquinolones, metronidazole, or doxycycline. The combination of amoxicillin/clavulanic acid, exerting bactericidal effects on proliferating bacteria, with tetracyclines, such as doxycycline, which inhibit the proliferation of bacteria, is not recommended in any case [23]. Doxycycline reduces the efficacy of penicillins [23]. The combination of two bactericidal antimicrobial substances may enhance the antibacterial effect against a broader spectrum of bacteria; however, synergistic effects of amoxicillin/clavulanic acid together with metronidazole or fluoroquinolones cannot be expected [24]. For example, dog-to-dog bite wounds are usually successfully treated with amoxicillin/clavulanic acid and require no additional combinations [25]. Since non-authorised combinations are frequently used according to our survey, guidance on indicated, safe, and effective use of non-fixed combinations of antibacterial drugs is desirable.

### 3.4. Prophylactic Use/Non-Antibacterial Use of Antibiotics

About 40% of the participants of the present survey stated that they had used ABs prophylactically in a small part of all patients. Prophylactic use was mainly in the context of surgical procedures to reduce postoperative complications. This is reasonable in surgeries with an increased risk of infection, e.g., in colorectal operations, pyometra, or oral cavity surgeries with a high bacterial preload, while not necessary as routine uses in “clean” surgeries [20,26,27]. According to Regulation (EU) 2019/6 (Article 107) [1], prophylactic use is allowed in individual animals when the risk of an infection or of an infectious disease is very high and the consequences are likely to be severe. Infection of the incised skin or soft tissues is a common but potentially avoidable complication of most surgical procedures [27]. The indiscriminate prophylactic administration of antibiotics (ABs) poses significant risks with respect to antimicrobial resistance (AMR) [28]. However, as previously reported, 32.1% of veterinarians in the UK even used ABs after scrotal castration [29]. In the present survey, the use of ABs in cases of castration was rare. Most participants stated that they administer ABs for specific types of surgery or circumstances, which are known to increase the risk for postoperative bacterial complications, such as long-lasting surgeries [27]. In general, antibiotics with a broad bactericidal spectrum and low toxicity such as amoxicillin should be injected at therapeutic doses prior to tissue incision, in order to receive the highest tissue levels during the operation and effective concentrations for a few hours after the surgery. To counteract AMR as a global threat, it should always be checked whether other measures, such as better hygiene management, can replace the use of ABs for prophylaxis [30].

The survey showed that ABs are also used for non-antibacterial purposes in a small number of patients for immunomodulation, as previously described [31,32], and against protozoan diseases, i.e., giardiasis or coccidiosis [33]. Indeed, several antimicrobial substances are effective against protozoa, such as metronidazole, which is authorised for the treatment of giardiasis in dogs. The use of ABs against AB-responsive diarrhoea, as stated by several participants, can be reasonable in cases of suspected giardiasis. Although effects against blood parasites are described [33,34,35,36], the use of antibiotics such as doxycycline against leishmaniasis, as some participants indicated, does not conform to AB guidelines [7] and Regulation (EU) 2019/6 [1]. This also applies to the use of ABs for other purposes, such as immunomodulation.

## 4. Materials and Methods

An anonymous, Germany-wide online survey was conducted to collect data on off-label use in small animal medicine. This quantitative cross-sectional study collected data once and, therefore, represents a meaningful snapshot [37,38].

### 4.1. Preliminary Work for the Survey

First, a table of preparations and active substances with the instructions for use was prepared in Excel (Microsoft^®^ Excel^®^ LTSC MSO (16.0.14332.20529) 64-bit), which formed the basis for the subsequent steps. The table contains all 105 AB-containing VMPs to be used systemically for dogs and 74 for cats from a total of 21 different active substances for dogs and 23 for cats that were authorised and available on the market in Germany at the time of the survey (13 July 2022; search conducted via VETIDATA). Drugs of different strengths were only counted once. ABs for local use were not taken into account. For systemically effective antimicrobial products, the following terms of authorisation stated in the SPCs were recorded: indication, dose, treatment frequency, route of administration, and treatment duration. The entire range of authorised routes of administration was taken into account using minimum and maximum values, e.g., an authorised dosage range of 8.75–25 mg/kg in relation to the total amount of active substances. In the later survey, all authorised routes of administration specifications were summarised as “as authorised: (…)”, and answer options deviating from this were arranged in equal gradations above and below; e.g., for amoxicillin/clavulanic acid, the dosages in relation to the total amount of active substances were as follows: “<4.38 mg/kg”, “4.38–8.74 mg/kg”, “as authorised: 8.75–25 mg/kg”, “25.1–37.5 mg/kg”, “37.6–50 mg/kg”, and “>50 mg/kg”.

In the course of developing the final survey, nine expert interviews were conducted in advance to identify questions on the topic of off-label use of ABs. They also served as a pre-test [37]. The findings and key topics were summarised [39] and then used to create a survey using the online survey tool Lime Survey, which was tested in a pilot run with a total of 25 veterinarians (from December 2022 to February 2023) with a focus on the treatment of cats and dogs. In addition to the actual thematic questions, the pilot survey also included so-called meta-questions. These check the content of the survey for the relevance of the questions and with regard to appropriateness, clarity, and comprehensibility in accordance with the “clinical sensibility testing” [37]. The meta-questions were taken from another study (https://www.cmaj.ca/content/suppl/2008/07/24/179.3.245.DC1, accessed on 16 April 2025) [37] and translated into German for this survey. The pilot survey was a quantitative survey. In the course of revising the pilot survey, answer options were added, questions were rephrased or made more specific, the arrangement of the questions and question blocks was partially changed, redundant questions were removed, and help texts and explanations were added.

### 4.2. Calls for Survey

The final survey (Appendix A) was announced via publications in journals that are read by the German veterinary profession, at veterinary congresses, and by contacting individual small animal veterinarians in Germany, which were randomly selected by using a Google Maps search.

**Figure 8:**
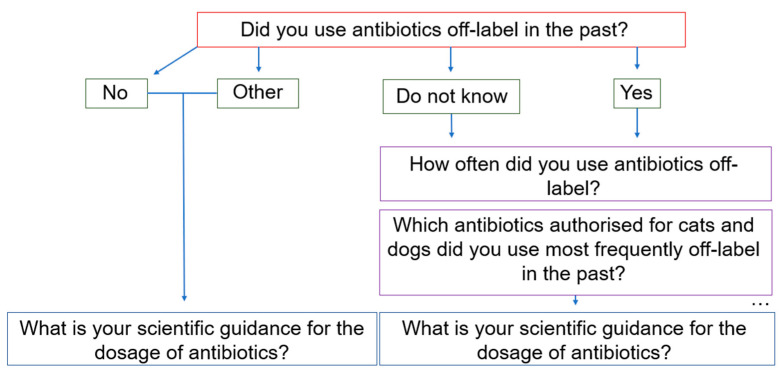
Decision tree: The figure shows an example of how the questions are linked depending on the answer options selected for previous questions. The backward navigation setting allowed the participants to change their answers retrospectively. This allowed “unsure” participants to select the “do not know” answer option and still continue to the follow-up questions. For example, by displaying the answer options of the follow-up question, the participants could gain certainty as to whether or not they had used antibiotics off-label. This provided the participants with the opportunity to continue on the “right path” in the survey.

Table 6 shows the arrangement of the question blocks with the number of questions. At the beginning of the questionnaire, participants were first asked whether they use ABs off-label at all. Depending on the answer, the participant automatically took a corresponding “path” in the course of the survey (Figure 8). The maximum number of questions was only reached if all conditional questions were “activated” by selecting a specific answer option (Figure 8). The third column of Table 6 shows the minimum number of questions if none of the conditional questions were “activated”. The penultimate row shows the number of detailed questions on the individual antibacterial preparations authorised for cats and dogs at the time the survey was created.

**Figure 9:**
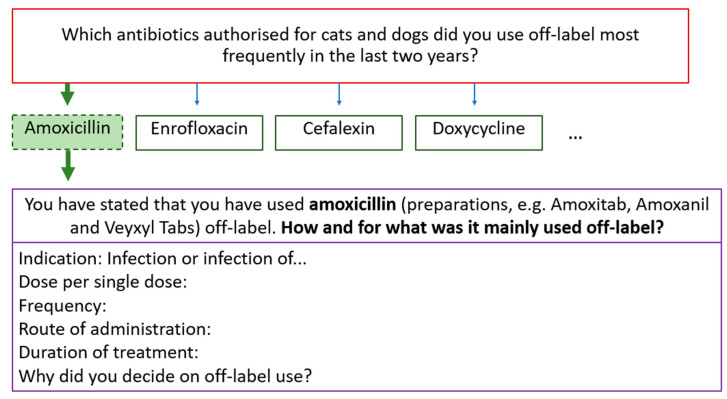
Decision tree of the detailed questions on active substances: If the answer option “Amoxicillin” (dashed green box) is selected for the top question, the respondent is redirected to a detailed question on the dosage and use of amoxicillin later in the survey (bottom two boxes with purple frame).

Detailed questions on individual active substances were only shown to participants who had selected them in a previous question (Figure 9). Therefore, the display of these detailed questions was dependent on the answer selection of a previous question. For example, if the active substance “Amoxicillin” (in the dashed box) is selected, the respondent will be shown the dosage question for “Amoxicillin” (purple-bordered questions) at the given time (Figure 9).

### 4.3. Testing for Representativeness and Evaluation

The survey period ran from the end of March 2023 to October 2023. To estimate the representative number, the total number of people to be considered for answering the survey was taken from the statistics on the veterinary profession in the Federal Republic of Germany for the year 2022 [40]. The figures from the statistics were taken from the category “Practitioners by animal species” from “Small animals”. Small animal practitioners from categories involving several animal species, e.g., “small animals and horses”, were not included. For small animals, this resulted in a population of 7358 veterinarians (practitioners: 5802, assistants: 1508, and practice representatives: 48). When calculating the sample size using the Epitool sample size calculator (https://epitools.ausvet.com.au/samplesize, accessed on 16 April 2025), with a confidence level of 95%, a margin of error of 5%, and a population size of 7358 veterinarians, the target size would be 310 participants. With 358 participants, i.e., every 5th small animal veterinarian in Germany, a representative sample was achieved due to the number of participants. According to different factors such as age, experience, professional expertise, or workplace, for example, the sample probably differs from the population. As there is a lack of comparable demographic data, it was not possible to compare the sample with the population.

The survey was analysed using the R programming language in the RStudio (RStudio 2023.09.1 + 494) interface. The raw data from Lime Survey were exported as a csv file and improvised in RStudio. Frequency tables were created during the evaluation. The graphs were created using Microsoft Excel (Microsoft^®^ Excel^®^ LTSC MSO (16.0.14332.20734) 64-bit).

## 5. Conclusions

As supported by this survey, in small animal medicine, the off-label use of antibiotics is common practice. In reasonable cases, this is in line with Regulation (EU) 2019/6 [1]. The responses concerning the impact of the new legal framework illustrated that veterinarians try to limit the off-label use of ABs as far as possible but that the well-being of the patients is their top priority. As stated in free-text comments, there is a constant conflict between the legal and practical requirements. Several participants criticised that many VMPs with the same active substances must be kept in stock in order to cover all important indications. As shown by this survey, there is a conflict between the prudent use of broad-spectrum cephalosporines (cefovecin) and the practicability of treatment, e.g., concerning cefovecin in cats. The therapeutic success of off-label AB uses largely met the expectations of the participants, and the majority of the veterinarians consider the success of AB therapy to be at risk or strongly at risk if they always had to strictly adhere to the terms of the authorisation. As indicated by this survey, amoxicillin/clavulanic acid VMPs, in particular, may require adjustments concerning doses and dosing intervals.

Although the survey is limited in terms of the number of questions and its representativeness of responses to single questions, it shows interesting data on the off-label use of ABs in dogs and cats. In summary, the risks of each off-label AB use need to be carefully balanced against the therapeutic advantages. However, this survey underlines that the existing legal opportunities, i.e., reclassification of ABs and off-label use, must be maintained.

The present survey should initiate more detailed studies in terms of clinical data, such as recovery data or complication rates, as well as the performance of bacteriological tests for reclassification of VMPs.

## Figures and Tables

**Table 1:** Percentage frequency of the participants who answered the question on off-label use of antibiotics authorised for cats and dogs (n = 330) with the number of authorised preparations (n). The type of off-label use concerned the indication in the sense of reclassification as well as the route of administration; higher dose (↑) or the frequency of administration (shortening of the dosing interval); in individual cases, the reduction in the dose (↓) and frequency (n), except for metronidazole, which was dosed lower by 31 participants, and the extension of the duration (↑); and in individual cases, the shortening of the treatment duration (↓). Other active substances with a low response rate were as follows: ampicillin (3), sulfadimidine (2), benzylpenicillin (1), lincomycin + spectinomycin (1), and sulfadimethoxine (1). * Incomplete query in the survey: marbofloxacin: frequency (cats) and duration (dogs) not queried; sulfadoxine + TMP: not queried for dogs, although authorised (1).

Antimicrobial Substances Authorised for Cats and Dogs	Absolute Frequency (Out of 330 Participants)	Type of Off-Label Use
**Amoxicillin/clavulanic acid (cats: 12, dogs: 12)**	226	Indication (151), Dose (↑ = 37), Frequency (↑ = 43, ↓ = 2) Duration (↑ = 29, ↓ = 16)
**Enrofloxacin (cats: 9, dogs: 14)**	128	Indication (54)
**Doxycycline (cats: 2, dogs: 2)**	94	Indication (cats: 54, dogs: 83) Duration (↑ cats = 19, dogs = 51)
**Metronidazole (cats: 4, dogs: 5)**	84	Duration (↑ = 38, ↓ = 2) Dose (↓ = 32, ↑ = 2) Route of administration (16)
Marbofloxacin (cats: 6, dogs: 8) *	79	Indication (cats = 50, dogs = 34) Dose (↑ cats = 18) Duration (↑ cats = 30)
**Cefovecin (cats: 1, dogs: 1)**	76	Indication (cats = 52, dogs = 19) Duration (↑ = 33)
**Amoxicillin (cats: 9, dogs: 16)**	59	Indication (37) Dose (↑ = 20) Duration (↑ = 20, ↓ = 4)
**Clindamycin (cats: 3, dogs: 10)**	48	Indication (cats = 22, dogs = 14) Duration (↑ cats = 14, dogs = 1; ↓ cats = 7, dogs = 7)
**Cefalexin (cats: 6, dogs: 9)**	36	Indication (cats = 13, dogs = 12)
**Sulfadiazine + trimethoprim (TMP) (cats: 1, dogs: 3)**	20	Duration (↑ cats = 7, dogs = 12) Indication (cats = 9, dogs = 9) Dose (↑ = 3, ↓ = 2) Frequency (cats: ↑ = 4, ↓ = 1) Route of administration (cats = 6)
**Gentamicin (cats: 3, dogs: 3)**	19	Indication (dogs = 7) Frequency (↑ cats = 4, dogs = 4) Duration (↑ cats = 4, dogs = 3)
**Pradofloxacin (cats: 2, dogs: 1)**	19	Indication (cats = 12, dogs = 2) Duration (cats: ↑ = 8, ↓ =1)
**Oxytetracycline (cats: 1, dogs: 1)**	12	Indication (4) Duration (↑ = 7)
**Metronidazole + spiramycin (dogs: 4)**	11	Duration (↑ = 4) Indication (3) Frequency (↑ = 2)
**Sulfadoxine + TMP (cats: 2, dogs: 1)**	8	Route of administration (6) Duration (↑ = 5) Frequency (↑ = 4)
**Benzylpenicillin-procaine (cats: 3, dogs: 3)**	6	Duration (↑ = 3, ↓ = 2) Indication (5) Frequency (↓ = 2) Dose (↑ = 1, ↓ = 1) Route of administration (2)
**Lincomycin (cats: 2, dogs: 2)**	5	Indication (3) Duration (↓ = 2, ↑ = 1) Route of administration (2)

**Table 2:** Summary of the free-text comments on the reasons for the off-label use of cefovecin. Problems with patient and owner compliance in connection with cats were frequently expressed. Specifically, it was described that regular trapping of cats and the administration of tablets by owners is often not feasible. The content of this reason can be assigned to the predefined response option “administration difficulties”.

Free-Text Comments: Cefovecin—Reasons	Repetitions
**Administration difficulties or patient/owner compliance, especially with cats**	9×
**Longer treatment duration necessary/recurrence**	2×
**No sample can be obtained for bacterial examination**	1×

**Table 3:** Excerpt of the answers to the following question: “Which veterinary antibiotics that are not authorised for cats and dogs have you used in the last two years?” (for systemic treatment); absolute frequency, n = 330. Multiple-choice question.

Antimicrobial Substance	Absolute Frequency (Out of 330 Participants)
**Fusidic acid**	41
**Chloramphenicol**	40
**Tylosin**	32
**Florfenicol**	28
**Tetracycline**	22
**Ronidazole**	19
**Polymyxin B**	18
**Orbifloxacin**	17
**Cefquinom**	16
**Neomycin**	15

**Table 4:** Summary of the free-text responses in conjunction with the response option “Other” to the question “For which indication did you use antibiotics prophylactically?”, n = 139. Multiple-choice question.

Prophylactic Use of Antibiotics: Indications	Repetitions
**Operations** - **Postoperative/for specific surgeries** - **Bone surgery** - **Surgeries with bacterial contamination (e.g., pyometra, intestinal operations, and tooth restoration/extraction)** - **Long-lasting surgeries** - **Abdominal cavity surgeries** - **Castration**	36×
**Bite injuries**	7×
**Parasites (e.g., giardia), bloody diarrhoea in puppies**	7×
**Partner animals with infection**	2×
**Others (fever, impending heat in old moribund female dogs)**	1×

**Table 5:** The most popular non-fixed antibiotic combinations in absolute and relative frequency in relation to the number of participants, n = 258.

Non-Fixed Antibiotic Combinations	Absolute Frequency	Relative Frequency (%) in Relation to the Number of Participants, n = 258
**Amoxicillin/clavulanic acid +** **fluoroquinolone**	214	83
**Amoxicillin/clavulanic acid +** **metronidazole**	93	36
**Amoxicillin/clavulanic acid + doxycycline**	41	16
**Cephalosporins + fluoroquinolone**	25	10
**Penicillins + fluoroquinolone**	24	9
**Doxycycline + fluoroquinolone**	10	4
**Others**	24	9

**Table 6:** Overview of the arrangement of the question blocks and the number of questions.

Question Blocks	Number of Questions	
	Maximum	Minimum
**Demographic questions—participants**	8	8
**General questions—off-label use of ABs**	11	4
**Prophylactic use**	3	1
**Use for non-antibacterial purposes**	3	1
**Antibiotic combinations**	6	2
**Use of ABs authorised in humans**	4	2
**Therapy success**	3	3
**Detailed question—antibiotic substances**	158	-
**Total**	196	21

## Data Availability

The datasets used and/or analysed during the current study are available from the corresponding author upon reasonable request.

## Appendix A. Survey: Off-Label Use of Antibiotics in Small Animal Medicine

### Appendix A.1. Demographic Questions

1. Are you an employed or self-employed veterinarian?
2. Which animal species do you specialize in treating?
3. Which specialty do you feel you belong to?
4. What qualifications do you have?
5. When did you receive your license to practice veterinary medicine?
6. In which practice structure do you work?
7. Where is the practice/clinic located?
8. In which federal state is the practice/clinic located?

### Appendix A.2. General Questions

1. Did you use antibiotics off-label in the last two years?
  - How often did you use antibiotics off-label?
  - Which antibiotics authorised for cats and dogs did you use off-label most frequently in the last two years?
2. Which veterinary authorised antibiotics that are not authorised for cats and dogs did you use in the last two years?
3. For which infection-related indication did you use antibiotics off-label?
4. What were the most common reasons for off-label use of antibiotics?
5. What is your scientific guidance for the dosage of antibiotics?
6. Did anything change in your off-label use since the new legal situation, which has been in force since 28 January 2022 (see below), and if so, how?
7. Is the patient’s weight determined before treatment?
  - How is the weight determined?
  - When is the weight determined during treatment with antibiotics?

### Appendix A.3. Prophylaxis

1. Did you use antibiotics prophylactically?
  - How often did you use antibiotics prophylactically?
  - For which indication did you use antibiotics prophylactically?

### Appendix A.4. Use for Non-Antibacterial Purposes

1. Did you use antibiotics for non-antibacterial purposes?
  - How often did you use antibiotics for non-antibacterial purposes?
  - For which indication did you use antibiotics for non-antibacterial purposes?

### Appendix A.5. Use of Non-Authorised Antibiotic Combinations

1. Did you ever use two different veterinary antibiotics together that were not already contained in one drug by the manufacturer?
2. Did you ever use more than two veterinary antibiotics together that were not already included in one drug by the manufacturer?
  - How often did you use two or more veterinary antibiotics together that were not already included in one drug by the manufacturer?
  - Which veterinary antibiotic combinations did you use that were not already included in one drug by the manufacturer?
  - For which indication did you use veterinary antibiotic combinations of two or more antibiotics that are not already included in one drug by the manufacturer?
  - Why did you decide to use antibiotics off-label?

### Appendix A.6. Human Antibiotics

1. Did you reclassify antibiotic drugs from human medicine?
  - How often did you reclassify antibiotics from human medicine whose active substances are also contained in veterinary products?
  - For what reason did you reclassify antibiotics whose active substances are also contained in veterinary products?
2. Does a ban on the use of the following groups in veterinary medicine pose a therapeutic problem for your veterinary work?

### Appendix A.7. Therapeutic Success

1. How often did the therapeutic success of the off-label use of antibiotics meet your expectations?
2. Do you consider the success of antibiotic treatment to be at risk by the Article 106 (1) rule “Veterinary medicinal products shall be used in accordance with the terms of marketing authorisation”?
3. Does this legal innovation have an influence on your choice and use of antibiotic preparations compared to the time before the new regulation/before 28 January 2022?

### Appendix A.8. Detailed Questions on Antimicrobial VMPs Authorised for Cats and Dogs

Scheme: You have stated that you have used xy (preparations, e.g.,) off-label. How and for what was it mainly used off-label?

- Indication: Infection or infection of …
- Dose in relation to the total amount of active substance per single dose:
- Frequency:
- Route of administration:
- Duration of treatment:
- Why did you decide on the off-label use of xy?
